# Prognostic Value of GDF-15 in Predicting Prolonged Intensive Care Stay following Cardiac Surgery: A Pilot Study

**DOI:** 10.1155/2021/5564334

**Published:** 2021-06-15

**Authors:** Henry Barton, Elisabeth Zechendorf, Dirk Ostareck, Antje Ostareck-Lederer, Christian Stoppe, Rashad Zayat, Tim Simon-Philipp, Gernot Marx, Johannes Bickenbach

**Affiliations:** ^1^Department of Surgical Intensive Medicine and Intermediate Care, University Hospital RWTH Aachen, Aachen, Pauwelstrasse 30, 52074 Aachen, Germany; ^2^Department of Thoracic and Cardiovascular Surgery, University Hospital RWTH Aachen, Aachen, Pauwelstrasse 30, 52074 Aachen, Germany

## Abstract

**Introduction:**

Predicting intensive care unit length of stay and outcome following cardiac surgery is currently based on clinical parameters. Novel biomarkers could be employed to improve the prediction models.

**Materials and Methods:**

We performed a qualitative cytokine screening array to identify highly expressed biomarkers in preoperative blood samples of cardiac surgery patients. After identification of one highly expressed biomarker, growth differentiation factor 15 (GDF-15), a quantitative ELISA was undertaken. Preoperative levels of GDF-15 were compared in regard to duration of intensive care stay, cardiopulmonary bypass time, and indicators of organ dysfunction.

**Results:**

Preoperatively, GDF-15 was highly expressed in addition to several less highly expressed other biomarkers. After qualitative analysis, we could show that preoperatively raised levels of GDF-15 were positively associated with prolonged ICU stay exceeding 48 h (median 713 versus 1041 pg/ml, *p* = 0.003). It was also associated with prolonged mechanical ventilation and rates of severe sepsis but not with dialysis rates or cardiopulmonary bypass time. In univariate regression, raised GDF-15 levels were predictive of a prolonged ICU stay (OR 1.01, 95% confidence interval 1–1.02, and *p* = 0.029). On ROC curves, GDF-15 was found to predict prolonged ICU stay (AUC = 0.86, 95% confidence interval 0.71–0.99, and *p* = 0.003).

**Conclusion:**

GDF-15 showed potential as predictor of prolonged intensive care stay following cardiac surgery, which might be valuable for risk stratification models.

## 1. Introduction

Advances in surgical and medical techniques as well as innovation in intensive care treatment have reduced mortality during and after cardiac surgery [1]. Conversely, morbidity has increased, mainly due to increased utilization of cardiac surgery in the elderly and more vulnerable patients with increasing amounts of preexisting diseases leading to more complex intensive care treatment [2]. Prolonged stay in the intensive care unit (ICU) following cardiac surgery represents a significant burden of disease. Up to 26% of patients will spend more than 3 days in the ICU after cardiac surgery, which is in turn associated with organ dysfunction, prolonged mechanical ventilation, and thus impaired outcomes [3].

To overcome these circumstances, prediction models of prolonged ICU stay can be helpful and should be implemented for efficient use of ICU resources [4]. However, the current risk stratification models' predictive ability has not improved despite further inclusion of patient and disease characteristics [5]. A novel approach of improving these models could be the inclusion of biomarkers for preinterventional risk stratification. The use of established and emerging biomarkers, such as CRP and GDF-15, has shown significant promise as predictors of outcome in myocardial infarction and heart failure [6, 7]. Additionally, the measurement of biomarkers would be a reliable variable, i.e., not prone to be influenced by inaccurate medical history or clinical judgement. Whilst several biomarkers have been investigated for use as predictors of mortality and morbidity in cardiac surgery patients, no studies have considered their value in predicting length of stay on the ICU [8, 9]. They could be an additional tool to provide information for preoperative optimization and accurate prediction of postoperative outcomes in this group of vulnerable patients. Biomarkers, especially cytokines, can be used to show underlying physiological and pathophysiological processes. For instance, biomarkers are already widely used in nephrology to predict kidney failure [10].

The primary objective of this study was an exploration of novel cytokines for prediction of prolonged ICU length of stay (PICULOS) in preoperative blood samples of cardiac surgery patients. Subsequently, a further analysis of highly expressed cytokines and their relationship to PICULOS was undertaken. The secondary objective included determining the usefulness of highly expressed cytokines for predicting severe sepsis, length of mechanical ventilation, renal replacement therapy, delirium, and mortality.

## 2. Materials and Methods

### 2.1. Study Design and Patient Selection

This prospective observational study used an existing biobank of blood samples collected from cardiac surgery patients (Ethics Committee of the University Hospital Aachen, RWTH University, Aachen, Germany, reference number EK 151/09). The principal enrolment criterion was cardiac surgery including coronary artery bypass grafting (CABG), aortic valve or combined CABG/aortic valve operations (AVR) performed during cardiopulmonary bypass at the University Hospital Aachen between January 2017 and July 2017. Exclusion criteria were other types of cardiac surgery, incomplete medical records, and missing blood samples. All patients provided written informed consent, and their identifying information was removed prior to analysis. Blood samples were collected 1 day preoperatively, directly upon ICU admission, 24 and 48 hours postoperatively. After centrifugation at 4°C for 10 minutes, plasma samples were frozen at -80°C.

We defined prolonged intensive care length of stay (PICULOS) as a time period greater than 48 hours as other studies in cardiac surgery demonstrated recovery within 48 hours and showed development of complications thereafter [11]. Patient characteristics and clinical parameters were retrieved from an electronically patient data recording system (medico//s, Siemens, Germany) and from a patient data management system (IntelliSpace Critical Care and Anesthesia, ICCA Rev. F.01.01.001, Philips Electronics, The Netherlands). The definition of severe sepsis as outlined in the Third International Consensus Definitions for Sepsis and Septic Shock was used [12]. Postoperative delirium was defined by CAM-ICU [13]. Acute kidney failure was defined as stage 3 kidney injury following KDIGO guidelines [14]. EuroSCORE II was calculated using the online tool [15]. We randomly selected 4 patients who underwent a normal ICU stay as a control group (non-PICULOS), i.e., shorter than 48 hours, and another group of 4 patients who stayed longer than 48 hours on the ICU (PICULOS).

### 2.2. Cytokine Screening

A cytokine and chemokine detection array (Proteome Profiler™ Human XL Cytokine Array Kit, R&D Systems, Minneapolis, MN, USA) covering 105 cytokines was used to screen 4 randomly selected preoperative blood samples from patients with PICULOS. These were matched by 4 randomly selected non-PICULOS patients. The plasma samples were not pooled. After dilution and overnight incubation, the detection membrane was washed and a detection antibody was added. Strepatividin-HRP and chemiluminescent detection agents were applied, and the signal produced was captured. The mean spot density was measured using the ImageQuant TL software (Version 8.1, GE Healthcare). These values were normalized against a calibrated measurement described in the test kit instructions. After this measurement, we averaged the mean spot density of each cytokine within the groups to allow a comparison between PICULOS and non-PICULOS blood samples.

### 2.3. GDF-15 Measurements and Patient Selection for Further Quantitative Analysis

After identifying GDF-15 as one cytokine showing the most distinctive differences between groups, we performed a quantitative measurement. A further patient selection (*n* = 89) was performed for both PICULOS and non-PICULOS after expanding our exclusion criteria to patients with glomerular filtration rate (GFR) below 50 ml/min or any signs of inflammation as both of these conditions can also cause GDF-15 elevation [16, 17]. We randomly selected 12 patients form each group, resulting in 24 patients in total. The stored plasma was thawed and analyzed using a commercially available enzyme-linked immunosorbent assay (Duoset® ELISA development system, human GDF-15, catalogue number DY957, R&D Systems, Minneapolis, MN, USA) following the manufacturer's protocol. Due to expected levels of GDF-15 and the sensitivity of the test kit, the samples were diluted according to the manufacturer's instruction up to 1 : 50.

### 2.4. Statistical Analysis

Discrete variables are given as absolute number and percentages. Continuous variables are presented as median and interquartile range (IQR) due to the skewed distribution of most of the parameters and to facilitate comparison. Differences between groups were assessed using Mann-Whitney *U* test and chi-squared test where appropriate.

Receiver-operator characteristic (ROC) curve analysis was performed in order to assess the cut-off value of GDF-15 for PICULOS (i.e., the values with the maximum sum of sensitivity and specificity). Area under curve (AUC) was also derived.

The prognostic value of GDF-15 for predicting PICULOS was assessed by performing a univariate logistic analysis. A probability value of <0.05 was considered significant. Statistical analysis was performed in GraphPad Prism 7 (GraphPad Software, San Diego, CA, USA) and SPSS 25 (SPSS, Chicago, IL, USA).

## 3. Results

### 3.1. Cytokine Screening

**Figure 1:**
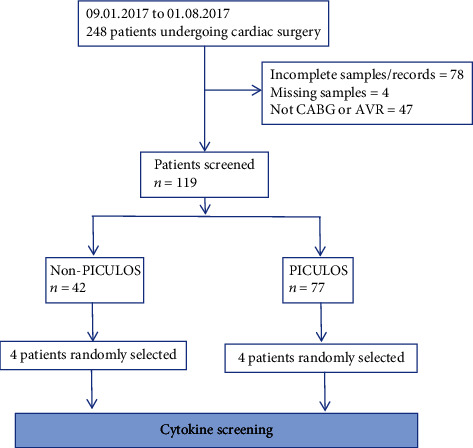
Patient selection. CABG: coronary artery bypass graft; AVR: aortic valve replacement; PICULOS: prolonged intensive care unit length of stay.

Of the 248 cardiac surgery patients for whom samples were stored in the biobank, 129 had to be excluded due to surgery procedures different from CABG or AVR or limited sample availability. In total, 119 patients could be included for cytokine screening (Figure 1). Amongst those screened, 42 patients spent less than 48 hours on the ICU, representing the non-PICULOS group, and 77 spent more than 48 hours on the ICU (PICULOS group). After randomly selecting 4 preoperative samples from the PICULOS group and another 4 control samples from the non-PICULOS group, we performed the cytokine screening with the Human XL Cytokine Array Kit.

**Figure 2:**
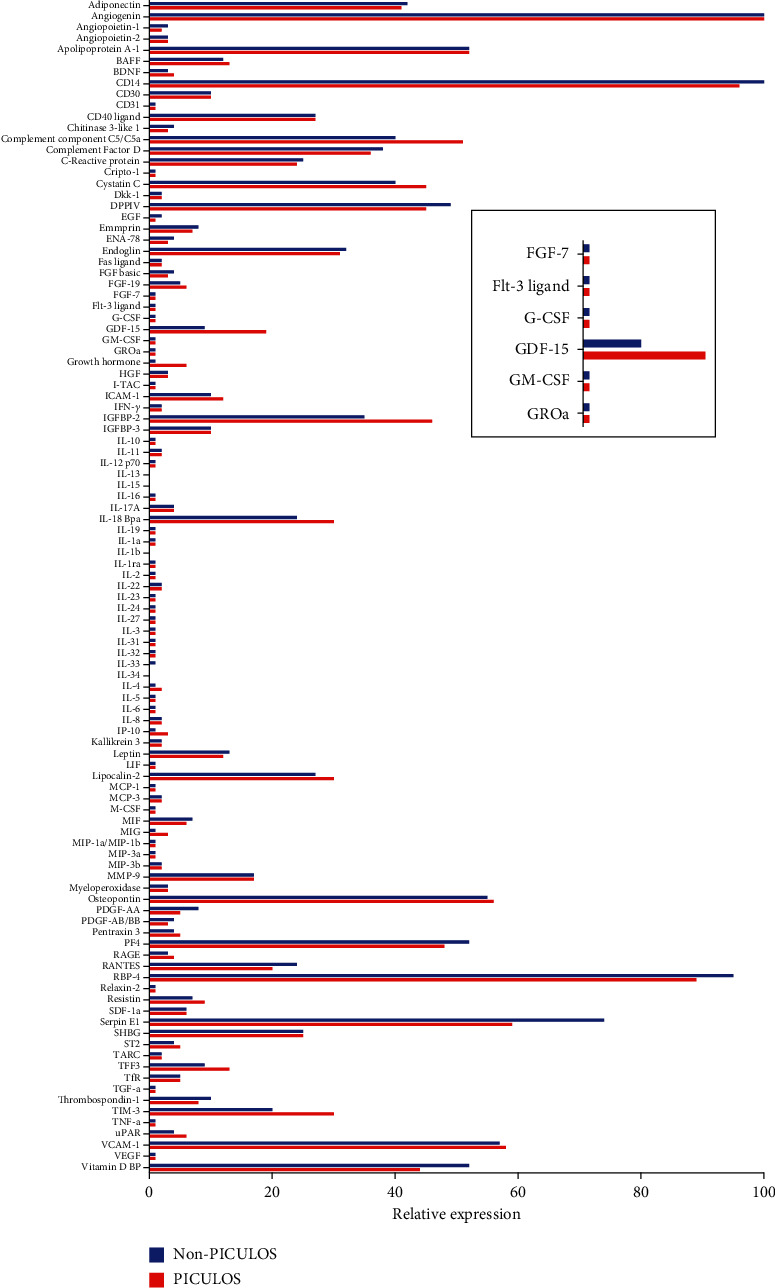
Analysis of the cytokine array profiler. The embedded image is a magnification of the greatest difference in cytokine expression.

**Figure 3:**
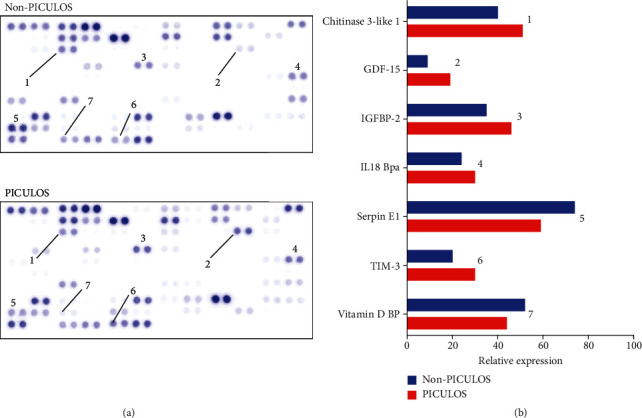
Two examples of completed cytokine array experiments, showing 105 different cytokines. The numbers below the spots in the left-hand graphic correspond to the respective cytokines in the right-hand bar chart.

We identified GDF-15 as a novel cytokine with higher preoperative expression in PICULOS patients after undergoing cardiac surgery. As depicted in Figure 2, in the PICULOS group mean GDF-15 expression was more than twice as high as in non-PICULOS patients. Other cytokines also showed a higher expression in the PICULOS group, especially Chitinase-3-like-1, IGFPB-2, IL-18 Bpa, and TIM-3, yet clearly less distinctive than GDF-15. Interestingly, Serpin-E1 and Vitamin D BP exhibited decreased expression. The other 98 cytokines were expressed at similar levels or not detectable. An example of both a PICULOS and non-PICULOS cytokine array with subsequent analysis is shown in Figure 3.

### 3.2. GDF-15 Measurements

#### 3.2.1. Patient Characteristics

For further quantification of the preoperative GDF-15 levels, GDF-15 serum levels from 12 patients with PICULOS and 12 non-PICULOS patients were analyzed. The median age was 67 for the non-PICULOS group and 79 for the PICULOS group, which was a statistically significant difference (*p* = 0.032). Additionally, EuroSCORE II was raised significantly in the PICULOS group with 3.85 percent versus 1.34 percent for the non-PICULOS cohort (*p* = 0.006). All other preoperative baseline characteristics showed no differences between the groups and are shown in Table 1.

The postoperative, during ICU stay, characteristics of the patients showed many significant differences which are described in Table 2. All patients in the PICULOS group had a significantly higher risk stratification score in SAPS II, Apache II, and SOFA. Also, the duration of mechanical ventilation was longer (8 vs. 200 hours, *p* = 0.001) as was the duration of vasopressor use (12 vs. 200 hours, *p* = 0.001). Severe sepsis was seen more frequently in the PICULOS group as was the need for dialysis and delirium. Interestingly, the duration of cardiopulmonary bypass does not affect the duration of ICU stay within the groups compared.

#### 3.2.2. GDF-15 and Outcomes

**Figure 4:**
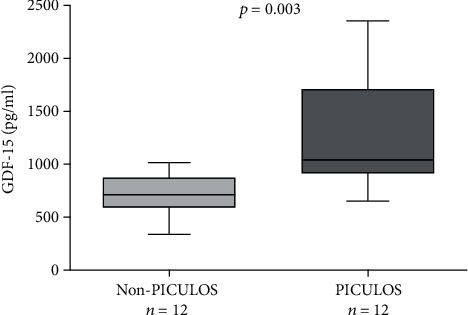
Preoperative plasma GDF-15 concentrations in patients undergoing cardiac surgery.

Concentrations of GDF-15 were raised in preoperative blood samples of PICULOS versus non-PICULOS patients, showing a significant increase within the PICULOS group (median 713 versus 1041 pg/ml, *p* = 0.003, Figure 4).

**Figure 5:**
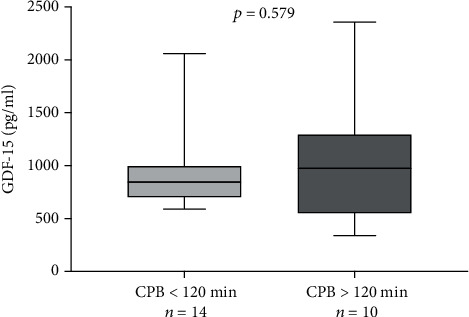
Preoperative GDF-15 vs. CPB time.

The most commonly performed surgery was coronary artery bypass graft. Also, 9 patients underwent a combined operation, whereby both a coronary bypass and aortic valve replacement were performed. The median time of cardiopulmonary bypass was 106 minutes for non-PICULOS patients versus 125 minutes for PICULOS patients. Raised levels of preoperatively raised GDF-15 were not associated with prolonged cardiopulmonary bypass duration (Figure 5).

**Figure 6:**
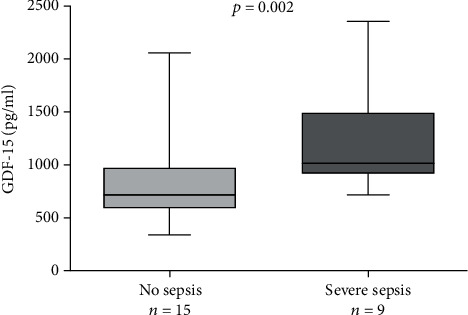
Severe sepsis rates compared to plasma GDF-15 levels.

Patients with preoperatively raised GDF-15 levels spent longer time undergoing mechanical ventilation. Regarding further clinical outcomes, patients with raised levels of GDF-15 required longer vasopressor therapy and were subject to severe sepsis more frequently as could be depicted in Figure 6. Rates of renal replacement therapy in the context with acute kidney failure were not increased with raised GDF-15 levels. Finally, rates of delirium were significantly associated with raised GDF-15 levels (median 718 versus 1491 pg/ml, *p* = 0.0006).

### 3.3. GDF-15 Prediction

As described in methods, we performed a logistic regression analysis of GDF-15 for prediction of prolonged ICU stay and also for other values. Univariate analysis showed GDF-15 levels (odds ratio 1.01, 95% confidence interval 1–1.02, and *p* = 0.029) to be predictive for a prolonged ICU stay. Additionally, age, EuroSCORE II, SAPS II, and SOFA scores were also prognostic for a prolonged ICU stay. However, when a multivariate analysis was performed, no further predictive value was found (Table 3).

In our population, preoperatively raised levels of GDF-15 were significantly better at predicting PICULOS than EuroSCORE II. ROC curve analysis of GDF-15 and PICULOS showed an AUC of 0.86 (95% confidence interval 0.71–0.99, *p* = 0.003) and a cut-off value of >905.8 pg/ml (sensitivity 83.33% and specificity 83.33%). An analysis of EuroSCORE II and PICULOS revealed an AUC of 0.81 (95% confidence interval 0.65–0.99, *p* = 0.008) with a cut-off value of 2.82% mortality (sensitivity 75% and specificity 75%).

## 4. Discussion

Our study is aimed at investigating potential novel cytokines predictive of prolonged ICU stay following cardiac surgery. We could demonstrate that cytokines are expressed differently in patients who spend longer than 48 hours on the ICU when compared to patients whose stay is shorter than 48 hours. Especially GDF-15 showed a significant, raised expression preoperatively in PICULOS patients versus non-PICULOS patient after both quantitative and qualitative analyses. Furthermore, severe sepsis rates, vasopressor support, and time of MV were significantly enhanced in the PICULOS group. Moreover, raised levels of GDF-15 were predictive of a prolonged ICU stay in univariate logistic regression. An increasing availability of cardiac surgery is counterbalanced by an increasing preexisting illness and frailty of patients. Moreover, ICU resources are scarce in most hospitals. To resolve this dilemma, better predictive models are required for sufficient risk stratification, especially in cardiac surgery patients. However, traditional preoperative risk stratification models such as the EuroSCORE II do not include any biomarkers. Other structural weaknesses are concerns about interobserver variability due to encoding mismatches or definition of risk factors [18] and methodological concerns regarding clinical validation [19]. Biomarkers, as indicators of biological stress, such as inflammation, can be used to predict clinical outcomes. They can be used to predict organ dysfunction, frailty, and biological aging. Therefore, inclusion of one or more biomarkers in risk stratification models could increase the accuracy and ease of use.

Generally, risk stratification using biomarkers has been sparingly evaluated in cardiac surgery. Prior research by Brown et al. [20] showed that inclusion of 4 additional biomarkers (cardiac troponin T, NT-ProBNP, and CRP) did not improve the predictive capability of a risk stratification model. Another study could show that brain natriuretic compound (BNP) can be used to predict postoperative mortality after cardiac surgery [21]. Raised levels of ST2, Galectin-3, and NT-ProBNP preoperatively were predictive of inhospital mortality in a paper by Polineni et al. [8]. Our study included CRP and ST2 during the cytokine screening process. CRP was highly expressed in all our patients, probably due to the high sensitivity of the cytokine array profiler and the multitude of organic reasons of heighted expression, both pathological and nonpathological. The other cytokine, ST2, was only marginally raised in the PICULOS group and therefore did not deserve further quantification. In sum, there exists a broad spectrum of biomarkers which have been evaluated regarding different clinical concerns. However, no specific biomarkers are described in terms of prolonged ICU stay after cardiac surgery.

Intensive care units provide high levels of complex and expensive care, especially after cardiac surgery. Many factors are associated with a prolonged, postoperative ICU stay. Subsequently, the Acute Physiological and Chronic Health Evaluation (APACHE) scoring system was revised to its latest version, APACHE IV. The APACHE IV uses 129 variables but no single biomarker to predict mortality rates and to estimate length of stay [22]. Additionally, the APACHE IV is designed to evaluate cardiac surgery patients. Other ICU prediction models such as SAPS2 [23] and SOFA [24] are used to solely predict ICU mortality and are therefore less useful to predict length of ICU stay. Both these models are calculated within 24 to 48 hours after admission to the ICU unit, respectively. An analysis of risk stratification models for prolonged ICU stay used a time frame between 6 to 48 hours as a “normal” ICU stay [25]. This study also showed that the various models of intensive care risk stratification (APACHE, SOFA, and SAPS) were inaccurate with poor predictive ability due to lacking validation and inadequate benchmarking.

Our study demonstrates that raised GDF-15 levels preoperatively are associated with prolonged ICU stay following cardiac surgery. GDF-15 has been analyzed extensively in medical practice as a marker of cardiac dysfunction [26]. In coronary artery disease patients, GDF-15 serum level was found to be significantly elevated compared to healthy controls [27]. It shows promise as a biomarker following ST-elevation acute myocardial infarction, predicting both short- and long-term outcomes [6]. Another recent study by Kuster et al. demonstrated that GDF-15 is useful in predicting middle term events in stable heart failure [7]. One previous study by Heringlake et al. could demonstrate that preoperatively raised levels of GDF-15 were an independent predictor of outcome following cardiac bypass surgery [9]. They showed that including preoperatively raised GDF-15 levels of over 1.8 ng/ml in the risk stratification model (EuroSCORE II) improved the predictive value, especially when compared to NT-ProBNP which did not result in reclassification. Further investigations by Guenancia et al. and Heringlake et al. could show that preoperatively raised GDF-15 levels were associated with acute kidney injury following CABG [28, 29]. Specifically, both studies showed prolonged ICU stay as a secondary outcome. These findings are in line with our results and underline the usefulness of GDF-15 in cardiac surgery patients/risk stratification. We could show that regardless of duration of cardiopulmonary bypass, GDF-15 levels were raised similarly. This suggests that the actual complexity of the cardiac surgery has no influence on preoperative GDF-15 levels.

The association of patient's outcome and raised levels of GDF-15 is not limited to the cardiac surgery and ICU setting. GDF-15, also known as MIC-1, is a stress-induced cytokine belonging to the superfamily of transforming growth factor-*β* (TGF-*β*) [30]. It is weakly expressed under all physiological conditions [31]. The normal range of GDF-15 has been reported as 150-1150 pg/ml [32] and 733-999 pg/ml [33]. Raised levels of GDF-15 are also measured in kidney failure [16] and various types of cancer such as colon [34], prostate [35], or melanoma [36].

Interestingly, we could demonstrate that GDF-15 showed significant differences in terms of length of ICU stay whereby higher levels of GDF-15 were predictive of more days on the ICU. It was also positively associated with significantly longer duration of mechanical ventilation. Furthermore, the rates of severe sepsis and vasopressor use were significantly higher in the patients with preoperatively raised GDF-15 levels. To our knowledge, this is the first study to show this association. However, we could not demonstrate that dialysis rates were increased, which is thought-provoking because GDF-15 is a biomarker for the prediction of kidney failure [37]. It was shown that preoperative GDF-15 is a biomarker of both renal dysfunction and muscle wasting in preoperative cardiac surgery patients which could in turn contribute to prolonged ICU stay [38].

Increased preoperative GDF-15 level might be indicative for an already existing cellular response to advanced inflammation [17]. In mice models, GDF-15 secreted by the myocardium was found to act protective and antihypertrophic [39]. Furthermore, after myocardial infarction, GDF-15 induction permitted infarct healing by limiting polymorphonuclear leucocyte (PMN) recruitment. Mechanistically, the anti-inflammatory effect of GDF-15 was caused by an interference with chemokine signaling [40].

Using univariate analysis, we demonstrated that GDF-15 levels are predictive of prolonged ICU stay. We also showed that EuroSCORE II, SOFA, and SAPS2 scores at ICU admission and age predicted prolonged ICU stay. Raised GDF-15 is associated with increasing age [41, 42]. We could confirm this finding in our observations. When we performed multivariate analysis, we could not demonstrate further predictive value, possibly due to the small sample size. Generally, as stated by Wiklund et al., GDF-15 is marker of all-cause mortality [43]. It is associated with age and many pathophysiological processes making it a rather unspecific marker of biological age and stress in humans.

## 5. Limitations

Our study has limitations that need to be addressed. The raised levels of GDF-15 in our patients could also be caused by other comorbidities, despite performing a selection. The influence of inflammation, kidney function, cardiovascular disease, and malignancy on the expression of GDF-15 remains unknown. There was also a difference in age between both groups which is a confounding factor especially given the fact that GDF-15 is raised in age. This influence is demonstrated in the lack of predictive ability following multivariate analysis. Generally, the low predictive ability in univariate logistic regression and the lack of predictive ability in multivariate regression are weaknesses of this study. The cause of this is the limited sample size. A further limitation is the prospective observational study character, yet with a retrospective analysis of data. It was performed in a single center without randomization. Also, we did not explore the long-term outcome of our patients. Finally, the data might not directly be transferable to other patient groups. However, we particularly focused on cardiac surgery, as this group is known to have a most pronounced perioperative risk.

## 6. Conclusion

We performed a broad explorative analysis of novel cytokines. This allowed us to exclude cytokines that showed no predictive value, but also identify cytokines which showed promise as novel biomarkers. We evaluated GDF-15 both qualitatively and quantitatively in regard to prolonged ICU stay and confirmed its predictive value in cardiac surgery patients. Our study is the first to demonstrate an association between preoperatively raised GDF-15 levels and prolonged ICU stay. Future research could include a further, prospective validation of GDF-15 as a predictor of prolonged ICU stay both in regard to specific groups such as cardiac surgery patients and the general population. Evaluating other clinical predictors and biomarkers such as BNP alongside GDF-15 would be a useful further study. The clinical utility of GDF-15 regarding positive and negative predictive values needs to be established for predefined length of stay. Also, further exploration of other raised or decreased cytokines could be performed in terms of risk stratification models. Finally, an evaluation in the form of randomized, prospective clinical trial to further asses GDF-15 as a predictive biomarker should be undertaken.

## Figures and Tables

**Table 1:** Baseline preoperative patient characteristics.

	Non-PICULOS *n* = 12	PICULOS *n* = 12	*p* value
Age, years	67 (53–75)	79 (77–83)	0.032
BMI, kg/m^2^	27 (25–32)	26 (25–29)	0.630
Gender (male/female)	7/5	7/5	1.000
Cardiac disease, *n*			0.856
(i) CHD	5	4	
(ii) Valvular	2	3	
(iii) Combined	5	5	
Comorbidity, *n*			
(i) Respiratory	3	1	0.333
(ii) Neurological	1	0	1.000
(iii) Renal	0	2	0.478
(iv) Diabetes	3	5	0.667
(v) Malignancy	0	3	0.217
Hemoglobin, g/dl	13.8 (12.9–15.1)	13.3 (11.9–14.4)	0.269
Leukocytes, /nl	7.0 (6.1–8.4)	7.4 (5.8–7.9)	0.884
CRP, mg/ml	1.5 (1.1–2.0)	3.8 (1.2–5.3)	0.113
Creatinine, mg/dl	0.99 (0.93–1.11)	1.04 (0.97–1.20)	0.200
GFR, ml/min/1.73 m^2^	74 (58–87)	60 (55–71)	0.068
EuroSCORE II, percent	1.34 (0.81–2.93)	3.85 (2.21–6.21)	0.007

Data are presented as median (IQR) or number (*n*). BMI: body mass index; CHD: coronary heart disease; CRP: complement reactive protein; GFR: glomerular filtration rate. Significance using chi-squared test or Mann-Whitney *U* test where appropriate.

**Table 2:** Postoperative, during ICU stay, patient characteristics.

	Non-PICULOS *n* = 12	PICULOS *n* = 12	*p* value
CPB, minutes	106 (84–132)	125 (89–155)	0.453
SAPS II at ICU admission	47 (40–48)	53 (50–56)	0.001
APACHE II at ICU admission	20 (18–22)	24 (21–24)	0.036
SOFA at ICU admission	8 (7–9)	10 (8-10)	0.016
LOS ICU, hours	27 (18–45)	424 (264–664)	0.001
Mechanical ventilation, hours	8 (6–12)	200 (24–447)	0.001
Vasopressors, hours	12 (7–21)	227 (81–439)	0.001
Severe sepsis, *n*	0	9	0.002
Delirium, *n*	0	7	0.046
Dialysis, hours	0	93 (82–183)	0.037
Death, *n*	0	2	0.478

Data are presented as median (IQR) or number (*n*). CABG: coronary artery bypass graft; CPB: cardiopulmonary bypass; LOS: length of stay; significance using chi-squared test or Mann-Whitney *U* test where appropriate.

**Table 3:** Results of univariable and multivariable logistic regression analyses for identifying predictors of prolonged ICU stay.

	Univariable logistic regression			Multivariable logistic regression		
	OR	95% CI	*p*	OR	95% CI	*p*
Age	1.17	1.02–1.34	0.025	1.132	0.91–1.47	0.260
Male	0.70	0.13–3.70	0.673			
BMI	0.97	0.81–1.13	0.653			
Diabetes	2.14	0.39–13.6	0.390			
GDF-15	1.01	1.00–1.02	0.029	1.003	0.99–1.01	0.461
GFR	0.93	0.86–1.00	0.073			
EuroSCORE II	1.80	1.12–3.59	0.043	1.149	0.48–2.79	0.738
CPB	1.01	0.99–1.03	0.370			
SAPS II	1.50	1.06–2.13	0.022	1.140	0.71–1.94	0.562
Apache II	1.41	1.03–2.10	0.050			
SOFA	2.21	1.02–4.80	0.045	1.590	0.52–8.04	0.463

BMI: body mass index; GFR: glomerular filtration rate; CPB: coronary pulmonary bypass; SOFA: sequential organ failure assessment.

## Data Availability

The datasets used and analyzed during the current study are available from the corresponding author on reasonable request.
